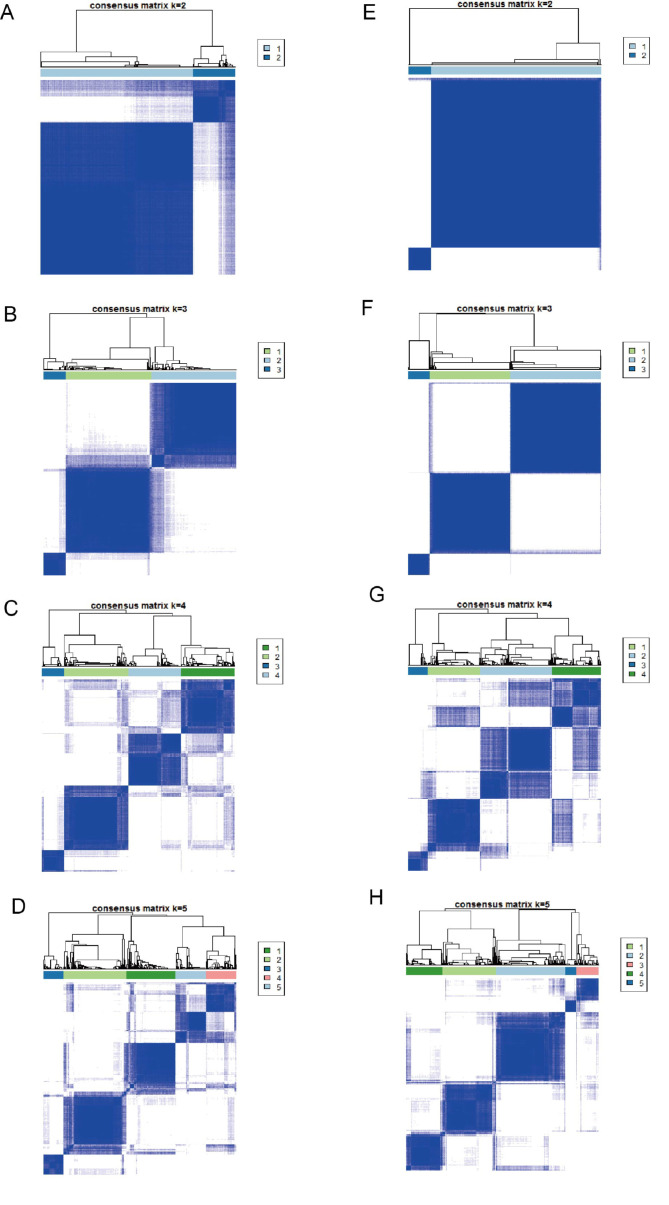# Identification of prognostic m^6^A modification patterns and score system in melanoma patients

**DOI:** 10.1097/MD.0000000000037950

**Published:** 2024-04-26

**Authors:** Feixiang Wang, Peijie Chen, Si Ouyang, Kaixin Xiong, Zichuan Liu, Yao Wang

**Affiliations:** a Medical Oncology Department, Affiliated Cancer Hospital & Institute of Guangzhou Medical University, Guangdong, Guangzhou, China.

**Keywords:** immunology, immunotherapy, m^6^A, melanoma, prognosis

## Abstract

N6-methyladenosine (m^6^A) is the most common modification on RNAs and LncRNAs. It plays an important role in cancer stem cell differentiation, T cell differentiation, and immune homeostasis. In this study, we explored the potential roles of m^6^A modification of RNA in melanoma and investigated the immune cell infiltration in tumor microenvironment in diverse m^6^Aclusters and different m^6^Ascore groups. A consensus clustering algorithm determined m^6^A modification patterns based on 14 m^6^A regulators, and further explored the biological functions and the connection with TME. An m^6^A-related gene signature (m^6^Ascore) was constructed based on m^6^A-related genes using principal component analysis. Three m^6^A modification patterns were identified based on 14 m^6^A regulators, named as m^6^Aclusters A-C. The prognosis of m^6^Acluster A was more favorable than m^6^Aclusters B and C, and it was more closely associated with immune regulation. To quantify the m^6^A modification patterns of individual tumor, an m^6^Ascore was constructed, and patients were classified into high and low m^6^Ascore groups. The low m^6^Ascore group, which had a favorable prognosis, was more relevant to immunology. The expression of PD-L1 was higher and the immunophenoscore (IPS) revealed stronger response to immunotherapy in the low m^6^Ascore group. This study identified 3 m^6^A modification patterns with different immune characteristics and constructed an m^6^Ascore system to predict prognosis and immunogenicity of patients, which is conducive to clinical prognosis judgment and individual treatment.

## 1. Introduction

Melanoma is a highly malignant cancer originating from melanocytes.^[1]^ Globally, there are about 232,100 new cases of cutaneous melanoma, and almost 55,500 deaths annually account for 0.7% of all cancer deaths.^[2]^ Most patients newly diagnosed at an early stage are suitable for surgical resection and have a favorable prognosis. The prognosis of advanced patients with metastatic disease is unsatisfactory under the conventional treatment, including surgical resection, chemotherapy, and targeted therapies.^[3]^ Previous studies demonstrated that melanoma is one of the most immunogenic tumors and has the greatest potential to respond to immunotherapy.^[4]^ Immune checkpoint inhibitors, including antiPD-1 and antiCTLA4 antibodies, have overturned the traditional treatment, and nearly 50% of patients are likely to experience tumor regression and long-lasting disease control, compared to <10% previously.^[5]^ To better improve the clinical application of immunotherapy, we need to understand more about the relationship between the individual tumor microenvironment (TME) and immunogenicity.

N6-methyladenosine (m^6^A) is the most common type of RNA methylation modifications. At present, m^6^A modification is widely detected in mRNA, lncRNA, and miRNA.^[6,7]^ Previous studies indicated that m^6^A modification is involved in a variety of biological processes, such as obesity, developmental defects, immunoregulation, and carcinogenesis.^[8,9]^ In immune regulation, m^6^A modification participates in immune recognition, innate immune response to viral infection, activation of adaptive immune response, and immune cell differentiation.^[10]^ Moreover, m^6^A plays a key role in TME, including the development of immune cells and stromal cells. Simultaneously, m^6^A is also regulated by various factors in TME, such as hypoxia and cellular stress.^[11]^ Therefore, m^6^A and TME mutually interact in tumor occurrence and progression.^[12]^ The dynamic function of m^6^A modification is mainly regulated by the modification proteins: methyltransferases (“writers”), demethylases (“erasers”), and binding proteins (“readers”).^[13]^ These regulators play indispensable roles in m^6^A modification, including RNA metabolism, processing, export, stability, and translation.^[13,14]^

Recently, numerous studies reported that most of these m^6^A regulators are frequently overexpressed in various cancer tissues, and some regulators promote tumor progression through demethylation.^[15–17]^ For example, methyltransferase-like 3 (METTL3) was upregulated in human melanoma tissues, and overexpression of METTL3 increased m^6^A activity, migration, and invasion of melanoma cells.^[18]^ Another study reported that fat mass and obesity-associated protein (FTO) could reduce the response to antiPD-1 inhibitor immunotherapy in melanoma patients.^[19]^ Here, we further explored m^6^A regulators to help identify novel therapeutic biomarkers and targets to develop effective treatment strategies for melanoma.

## 2. Materials and methods

### 2.1. Datasets of skin cutaneous melanoma

We downloaded the gene expression data and corresponding clinical annotation from publicly available datasets of the Cancer Genome Atlas (TCGA), Genomic Data Commons (https://portal.gdc.cancer.gov/) and Gene-Expression Omnibus database (GSE65904). Samples without survival information were eliminated from further analysis. Finally, a total of 668 patients were enrolled in our research, including 458 patients in TCGA and 210 patients in gene-expression omnibus, and each sample corresponded to 1 patient. Data collection was performed in August 2021.

### 2.2. Consensus cluster of 14 m^6^A regulators

We sorted out 19 m^6^A regulators for our study according to the previous research.^[16,20–22]^ These 19 m^6^A regulators included 7 writers (METTL3, METTL14, METTL16, WTAP, ZC3H13, RBM15, RBM15B), 10 readers (YTHDC1, YTHDC2, YTHDF1, YTHDF2, YTHDF3, HNRNPC, LRPPRC, HNRNPA2B1, RBMX, ELAVL1), and 2 erasers (FTO, ALKBH5). The RCircos R package was used to show the copy number variation (CNV) location of 19 m^6^A regulators on 23 pairs of chromosomes.^[23]^ Then Kaplan–Meier analysis evaluated the relationship between the prognosis of melanoma and 19 m^6^A regulators, which found 14 m^6^A regulators were associated with the prognosis of melanoma. Based on the expression of 14 m^6^A regulators, we performed unsupervised clustering analysis to identify distinct m^6^A modification patterns. The above steps were run by the Consensus Cluster Plus package and were repeated 500 times at least to ensure the stability of classification. The number of clusters was determined by the dispersion, cophenetic, and silhouette coefficients.

### 2.3. Gene set variation analysis (GSVA)

GSVA enrichment analysis with the “GSVA” R package was performed to identify the difference on biological processes and pathways among the m^6^A modification patterns. The gene sets of “c2.cp.kegg.v7.4.symbols” downloaded from MSigDB database was used for running GSVA analysis. Gene ontology (GO) and Kyoto Encyclopedia of Genes and Genomes (KEGG) enrichment analyses for m^6^A phenotype-related genes were performed in clusterProfiler R package with the cutoff value of false discovery rate (FDR) < 0.05.

### 2.4. ssGSEA on immune cell infiltration estimation

Single sample gene set enrichment analysis (ssGSEA) was carried out to quantify the relative abundance of each immune cell infiltration in TME of 3 modification patterns and m^6^Ascore groups. The enrichment scores were calculated by ssGSEA analysis and represented the relative abundance of each immune cell in TME in each sample.

### 2.5. Identification of DEGs between m^6^A distinct patterns

We identified m^6^A-related differentially expressed genes (DEGs) among the 3 m^6^A modification patterns with “limma” R package. An adjusted *P* value <.001 (*P* < .001) was set for the significance filtering criterion of DEGs.

### 2.6. Generation of m^6^Ascore

We constructed a scoring system to quantify the m^6^A modification patterns of individual tumor – an m^6^A gene signature, which was called the m^6^Ascore. The steps for establishing m^6^A gene signature were as follows:

We extracted the overlapping DEGs identified from 3 m^6^Aclusters. A univariate cox regression model was used to identify genes associated with the prognosis of melanoma among overlapping DEGs. The genes associated with significant prognosis were screened out for further analysis. Then based on the expression data of prognostic genes, patients were classified into several groups through unsupervised clustering. The consensus clustering algorithm determined the number (n = 3) and the stability of gene clusters. According to the expression of prognostic genes, we constructed m^6^A gene signature using principal component analysis; principal component 1 and 2 were both selected as signature scores. We then figured out a formula according to previous research: m^6^Ascore= ∑(PC1i + PC2i),^[24,25]^ where “ i ” represents the expression of significant m^6^A phenotype-related genes. Based on the median score, we divided the patients into high and low m^6^Ascore groups.

### 2.7. Correlation between m^6^Ascore and immunity

To identify the association between the m^6^Ascore and TME, we performed a correlation analysis between m^6^Ascore and 23 types of immune cell infiltration. The immunophenoscore (IPS), a quantification of determinants of tumor immunogenicity, is a superior predictor of response to the immune checkpoint blockade in melanoma.^[26]^ The scoring scheme consists of 4 categories of immune-related genes: MHC molecules, immunomodulators, effector cells, and suppressor cells. The weighted average Z-score is calculated from the average z-score of the samples of the 4 categories within the category, and the sum of the weighted average Z-score is IPS.

### 2.8. Statistical analysis

The Kaplan–Meier (KM) method was applied to draw the survival curves for the prognostic analysis, and log-rank tests were applied to ascertain significance of differences. A univariate Cox regression model was utilized to calculate the hazard ratios for m^6^A regulators and m^6^A phenotype-related genes. The multivariate Cox regression model was used to determine the independent prognostic factors. The mutation prospect of high and low m^6^Ascore in patients was presented by the maftools package with waterfall function. The statistical analyses in this study were performed by R software (R version 4.1.0). All 2-sided *P* values <.05 (*P* < .05) were considered statistically significant.

## 3. Results

### 3.1. Characteristics of melanoma patients

**Figure 1.**
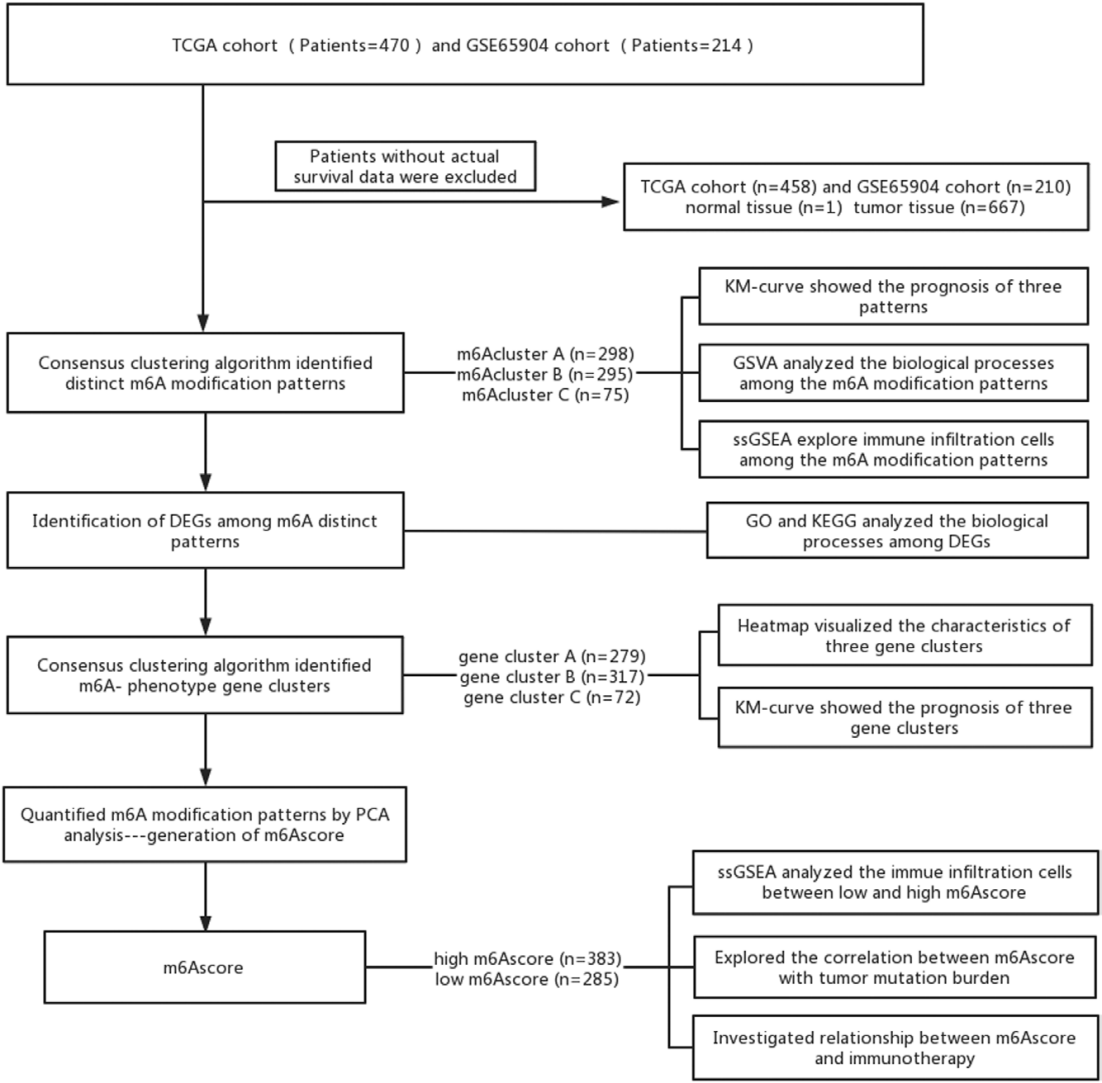
Workflow of this study.

The TCGA cohort with 470 samples and the GSE65904 cohort with 214 samples were downloaded for our study. Samples without survival information were eliminated from further analysis. In total, 668 patients with melanoma were enrolled in our study, including 458 patients from TCGA and 210 patients from the GSE65904 dataset, with an average age of 59.5 years (15–91 years). The clinicopathological characteristics of these patients are shown in Table 1 and 2. The design process of our study is shown in the flow chart (Fig. 1).

**Table 1:** Characteristics of 458 patients with melanoma in TCGA.

Characteristics	N(%)
Age	
≤65	296 (64.6)
>65	162 (35.4)
Gender	
Male	284 (62.0)
Female	174 (38.0)
Stage	
I/II NOS	10 (2.2)
Stage 0	6 (1.3)
Stage I	76 (16.6)
Stage II	139 (30.3)
Stage III	169 (36.9)
Stage IV	22 (4.8)
Unknown	36 (7.9)
T	
T0	23 (5.0)
T1	41 (9.0)
T2	76 (16.6)
T3	89 (19.4)
T4	151 (33.0)
Tis	7 (1.5)
TX	44 (9.6)
Unknown	27 (5.9)
M	
M0	409 (89.3)
M1	23 (5.0)
Unknown	26 (5.7)
N	
N0	228 (49.8)
N1	73 (15.9)
N2	49 (10.7)
N3	54 (11.8)
NX	35 (7.6)
Unknown	19 (4.1)

TCGA = the cancer genome atlas.

**Table 2:** Characteristics of 210 patients with melanoma in GSE65904.

Characteristics	N(%)
Age	
≤65	116 (55.2)
>65	93 (44.3)
Unknown	1 (0.5)
Gender	
Male	124 (59.0)
Female	86 (41.0)
Stage	
Stage I–II	49 (23.3)
Stage III–IV	154 (73.3)
Unknown	7 (3.3)

### 3.2. Landscape of genetic variation of m^6^A regulators in melanoma

**Figure 2.**
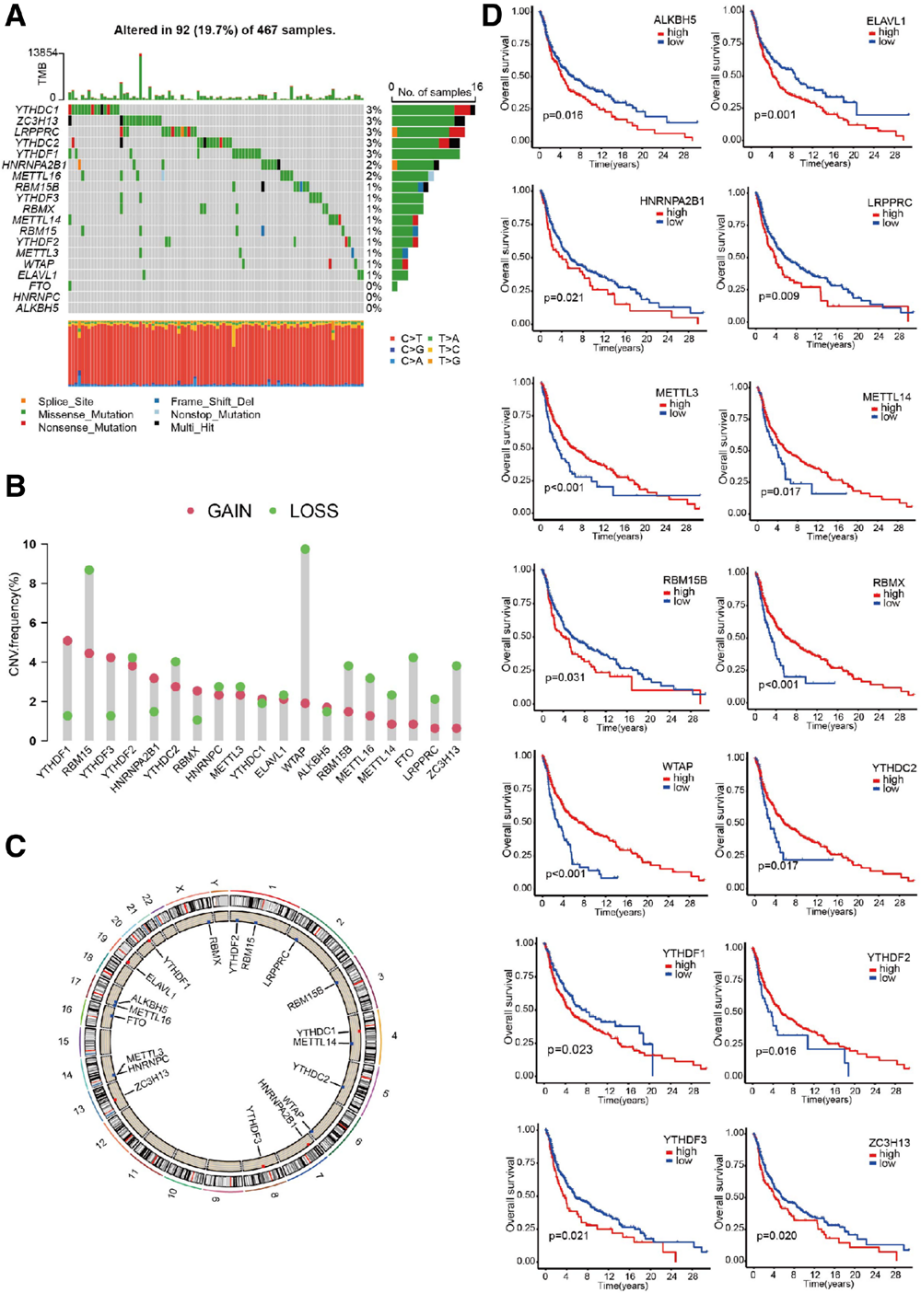
Genetic variation of m^6^A regulators in melanoma and their effect on prognosis. (A) Summary of the somatic mutations and copy number variations of 19 m^6^A regulators in 467 melanoma patients (TCGA-SKCM mutation data). (B) The copy number variation (CNV) frequency of m^6^A regulators in the TCGA cohort. Green dots: deletion frequency. Red dots: amplification frequency. (C) The location of CNV alterations of m^6^A regulators on 23 chromosomes from TCGA cohort. (D) The KM-curve of 14 m^6^A regulators with significant prognosis in 668 melanoma patients (TCGA and GSE65904 cohort). KM = Kaplan-Meier, m6A = N6-methyladenosine, TCGA = the cancer genome atlas.

In this study, a total of 19 acknowledged m^6^A regulators including 7 writers, 10 readers and 2 erasers from the previous literature review^[16,20–22]^ were enrolled for analyses. We first made a summary of the somatic mutations and copy number variations of 19 m^6^A regulators in melanoma. Mutations occurred in 92 of the 467 samples from the TCGA melanoma mutation database. The highest mutation frequency was 3%, including YTHDC1, ZC3H13, LRPPRC, YTHDC2 and YTHDF1; whereas FTO, HNRNPC, and ALKBH5 did not show any mutations in the melanoma samples (Fig. 2A). Frequency analysis of 19 m^6^A regulators presented a prevalent alteration in CNV. More than half of the alteration in m^6^A regulators showed deletion in copy number; especially RBM15, WTAP, FTO and ZC3H13 had a widespread CNV deletion, while YTHDF1 and YTHDF3 displayed prevalent CNV amplification (Fig. 2B). Location of the CNV alterations of m^6^A regulators on chromosomes is shown in Figure 2C. The KM curve revealed that 14 of the 19 m^6^A regulators were closely associated with prognosis of patients with melanoma (*P* < .05, Fig. 2D). Overexpression of METTL3, METTL14, RBMX, WTAP, YTHDC2, and YTHDF2 showed positive correlation with survival. In contrast, overexpression of ALKBH5, ELAVL1, HNRNPA2B1, LRPPRC, RBM15B, YTHDF1, YTHDF3, and ZC3H13 showed negative correlation with survival. These 14 regulators were selected for further study.

### 3.3. Generation of m^6^A modification patterns

**Figure 3.**
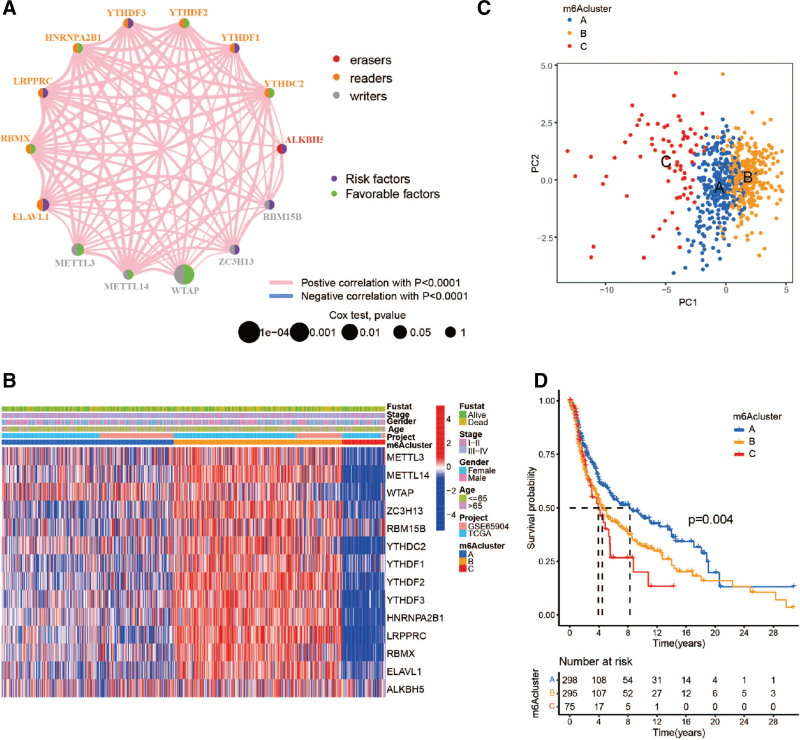
Three m^6^A methylation modification patterns based on 14 m^6^A regulators. (A) The interaction between 14 m^6^A regulators in melanoma. The calculated values of log-rank test were *P* < .0001, *P* < .001, *P* < .01, *P* < .05, and *P* < 1, respectively. Green dots: favorable factors. Purple dots: risk factors. (B) Unsupervised clustering of 14 m^6^A regulators in the melanoma cohorts, named as m^6^Aclusters (A–C) (TCGA and GSE65904). (C) Principal component analysis performed on the transcriptome profiles of 3 m^6^A modification patterns. (D) Survival analyses for the 3 m^6^A modification patterns based on 668 patients with melanoma, including 298 samples in m^6^Acluster-A, 295 samples in m^6^Acluster-B, and 75 samples in m^6^Acluster-C (*P* = .004). m^6^A = N6-methyladenosine, TCGA = the cancer genome atlas.

**Figure 4.**
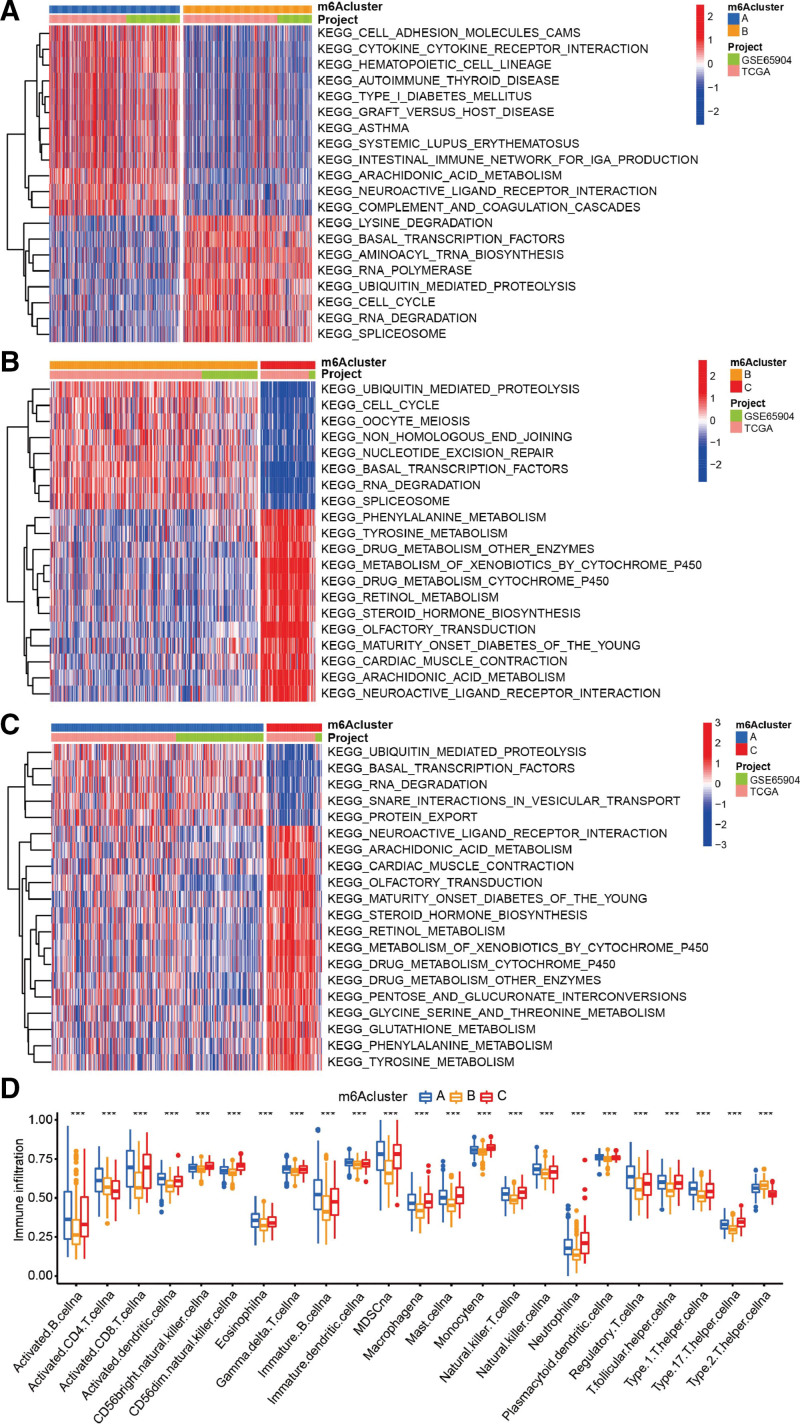
Characteristics of 3 m^6^A modification patterns. (A–C) Biological functions and pathways in 3 m^6^A modification patterns shown by GSVA enrichment analysis. These biological processes were visualized by heat map, with red representing activated pathways and blue representing inhibited pathways. The melanoma cohorts (TCGA and GSE65904) were used as the patients’ annotations. (A) m^6^Acluster A vs m^6^Acluster B; (B) m^6^Acluster B vs m^6^Acluster C; (C) m^6^Acluster A vs m^6^Acluster C. (D) The abundance of immune infiltrating cells in these 3 m^6^A modification patterns. Statistical *P* value was indicated by the asterisks (*P < .05, **P < .01, ***P < .001). m6A = N6-methyladenosine, TCGA = the cancer genome atlas.

A comprehensive landscape of the interaction and prognostic significance of 14 m^6^A regulators in melanoma patients is illustrated in the m^6^A regulator network (Fig. 3A). Based on the expression of 14 m^6^A regulators, patients were classified into 3 modification patterns by unsupervised clustering (Supplemental Digital Content (Figure S1, http://links.lww.com/MD/M303), A-D), including 298 cases in cluster A, 295 cases in cluster B, and 75 cases in cluster C (Fig. 3B). The principal component analysis diagram displayed significant differences in the expression profile of m^6^A regulators within the 3 modification patterns (Fig. 3C). Compared with cluster B and C, cluster A had a significant survival advantage (Fig. 3D). Additionally, GSVA showed that immune-related pathways such as cytokine-cytokine receptor interaction, autoimmune thyroid disease, graft-versus-host disease, asthma, and systemic lupus erythematosus were mainly enriched in m^6^Acluster A (Fig. 4A and C). While m^6^Acluster B was associated with pathways of ubiquitin mediated proteolysis, cell cycle and RNA degradation (Fig. 4A and B). And m^6^Acluster C was related to phenylalanine, drugs, retinol, tyrosine and some other metabolic pathways (Fig. 4B and C). Subsequently, the analysis of TILs (tumor infiltrating lymphocytes) showed that cluster-A had abundant immune cell infiltration, including B, CD4 T, dendritic, gamma delta T, and natural killer cells (Fig. 4D).

### 3.4. Analysis of DEGs between different m^6^A modification patterns

**Figure 5.**
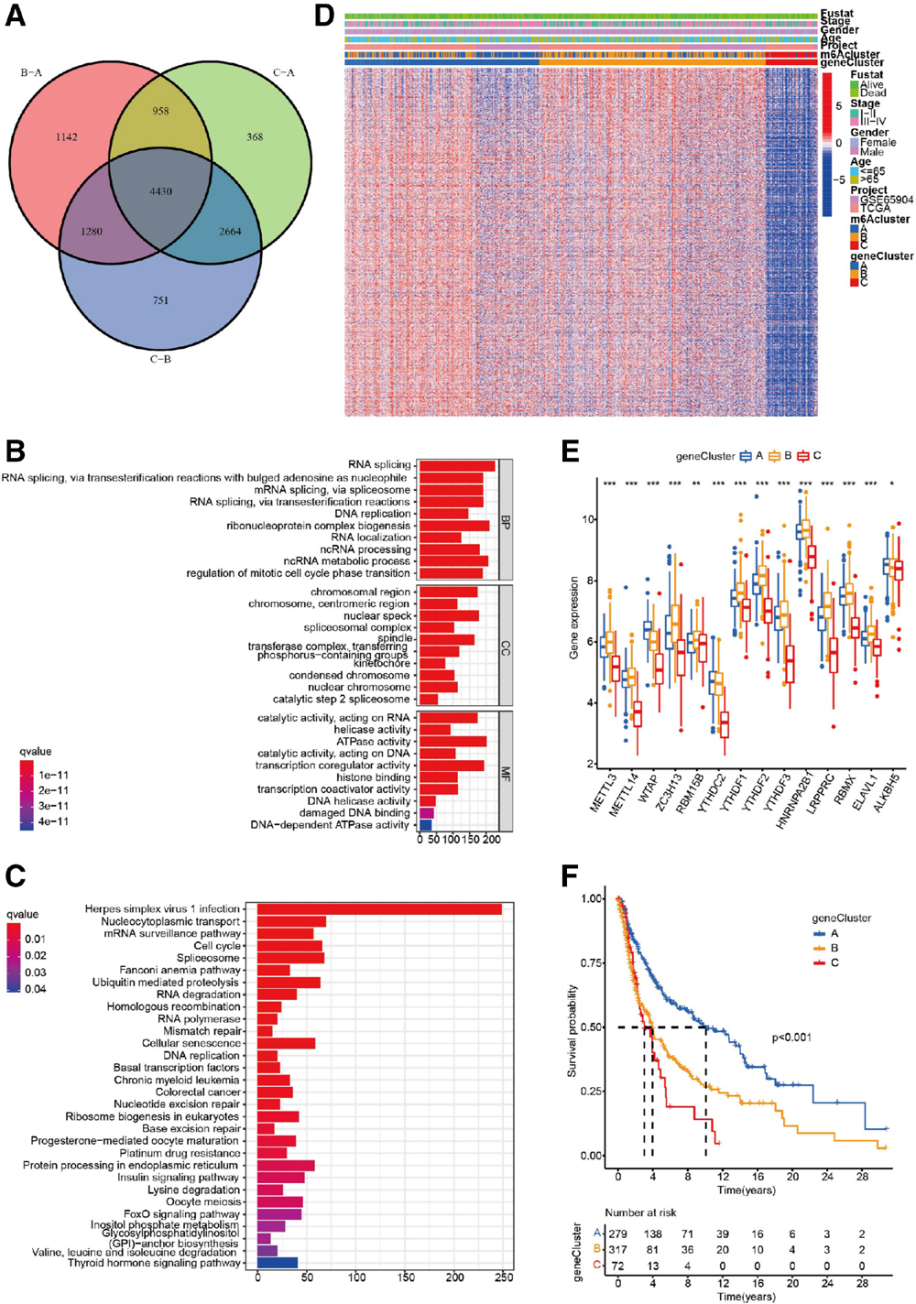
m^6^A modification pattern-related DEGs in melanoma. (A) A total of 4430 m^6^A modification pattern-related DEGs are shown by the Venn diagram. (B-C) GO and KEGG enrichment analysis show the functional annotation of m^6^A-related genes. (D) Unsupervised clustering of overlapping m^6^A-related genes classify patients into different genomic subtypes, named as m^6^A gene clusters (A-C) (E) The expression of 14 m^6^A regulators in 3 gene clusters. Statistical *P* value was indicated by the asterisks (*P < .05, **P < .01, ***P < .001). (F) Survival analyses for the 3 m^6^A modification genomic phenotypes based on 668 patients with melanoma, including 279 samples in gene cluster-A, 317 samples in gene cluster-B, and 72 samples in gene cluster-C (*P* < .001). DEGs = differentially expressed genes, GO = gene ontology, KEGG = Kyoto encyclopedia of genes and genomes, m6A = N6-methyladenosine.

To elucidate further the underlying biological behavior of each m^6^A modification pattern, we identified 4430 DEGs based on the m^6^A regulator expression (Fig. 5A). GO enrichment analysis illustrated that these DEGs were related to RNA splicing, RNA localization, and transcription coregulator activity (Fig. 5B). KEGG enrichment analysis displayed that these genes were associated with viral infection, mRNA surveillance pathway, the cell cycle, and chronic myeloid leukemia (Fig. 5C). We then screened out 845 genes associated with the prognosis of melanoma among overlapping DEGs for further analysis. Based on these overlapping prognostic genes, patients were classified into 3 m^6^A modification genomic phenotypes by unsupervised clustering (Supplemental Digital Content (Figure S1, http://links.lww.com/MD/M303), E-H), including 279 cases in gene cluster A, 317 cases in gene cluster B and 72 cases in gene cluster C (Fig. 5D). The heatmap displayed the characteristics of each patient in the m^6^A gene cluster (Fig. 5D). The expression of m^6^A regulators was significantly different in the 3 m^6^A gene clusters (Fig. 5E). The prognostic analysis of the 3 gene subtypes showed that m^6^A gene cluster A had a better survival advantage than m^6^A gene clusters B and C. (Fig. 5F).

### 3.5. Generation of m^6^Ascore

**Figure 6.**
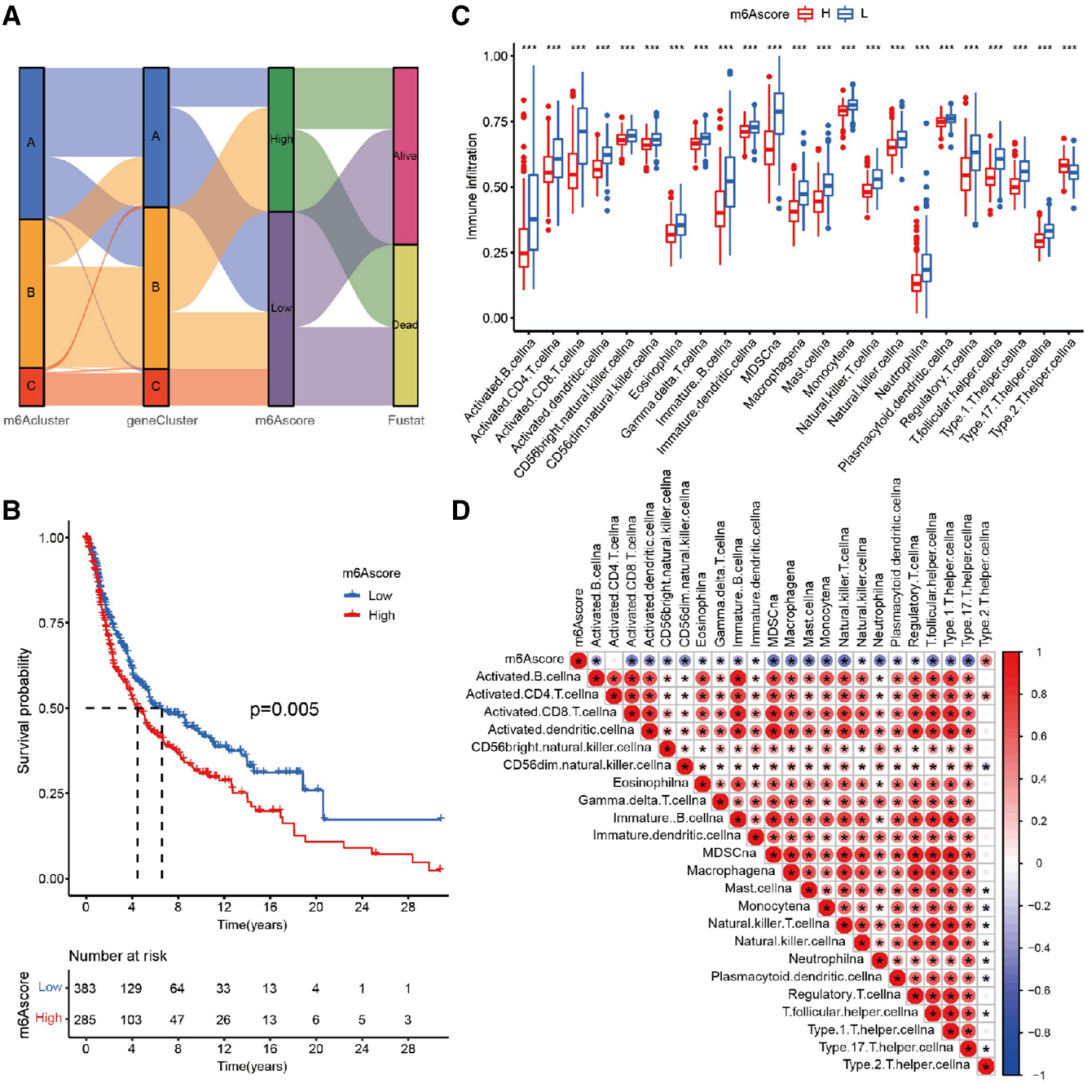
Construction of the m^6^Ascore. (A) Alluvial diagram shows the changes of m^6^Acluster, gene cluster, m^6^Ascore, and survival status. (B) Survival analyses for the low (383 samples) and high (285 samples) m^6^Ascore groups based on 668 patients with melanoma (*P* = .005). (C) The abundance of immune infiltrating cells in low and high m^6^Ascore groups. Statistical *P* value is indicated by the asterisks (*P < .05, **P < .01, ***P < .001). (D) Correlations between m^6^Ascore and 23 types of known immune infiltrating cell cells. Positive correlation is shown in red and negative correlation is shown in blue. m6A = N6-methyladenosine.

The above analyses indicated that m^6^A modification could play crucial part in immune regulation and prognosis of melanoma, but these results are based only on the patient population. To predict accurately the m^6^A modification pattern of individual patient with melanoma, we needed to consider individual heterogeneity and complexity. Therefore, based on the aforementioned prognostic m^6^A pattern-related DEGs, a scoring system termed the m^6^Ascore was constructed. The attribute changes of individual patient were visualized by an alluvial diagram (Fig. 6A). Compared with the high m^6^Ascore group, patients with a low m^6^Ascore were significantly associated with better survival (Fig. 6B). We further investigated the infiltration of immune cells in the 2 groups. The differential analysis of immune cell infiltration confirmed that the low m^6^Ascore group had abundant immune cell infiltration compared to the high m^6^Ascore group (Fig. 6C). Most of these 23 types of infiltrated immune cells significantly correlated with the m^6^Ascore, including B cells, CD8 T cells, natural killer cells, eosinophils, MDSCs (myeloid-derived suppressor cells), macrophages, and regulatory T cells (Fig. 6D).

### 3.6. Immunotherapeutic characteristics of high- and low-m^6^Ascore groups

**Figure 7.**
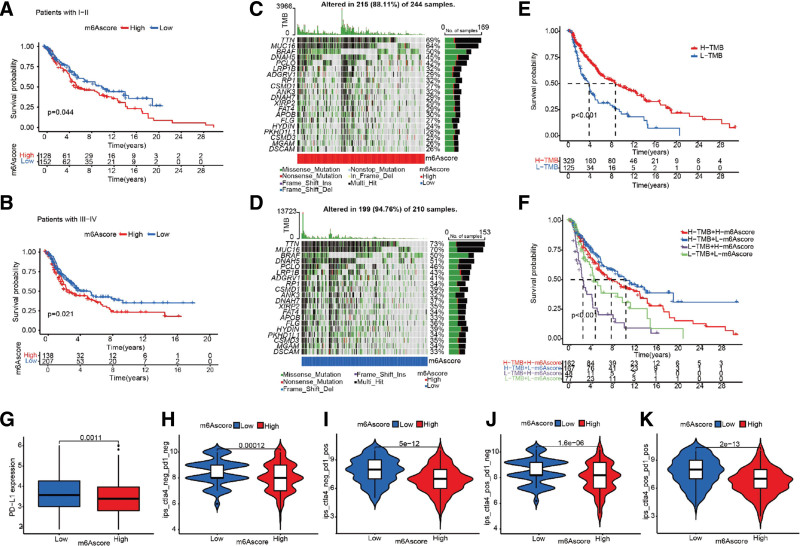
Immunotherapeutic characteristics of the m^6^Ascore. (A-B) Survival analyses for patients with low and high m^6^Ascore in different tumor stages (A) patients with I to II, *P* = .044; (B) patients with III-IV, *P* = .021). (C-D) The waterfall plot of tumor somatic mutation in patients with high m^6^Ascore (C) and low m^6^Ascore (D). (E) K-M curve of patients with high or low TMB (*P* < .001). (F) K-M curve of patients with high (or low) TMB and high (or low) m^6^Ascore (*P* < .001). (G) The expression of PD-L1 in low and high m^6^Ascore groups (*P* = .0011). (H-K) Immunophenoscore (IPS) comparison between low and high m^6^Ascore groups in melanoma patients in the CTLA4 negative/positive or PD-1 negative/positive types. CTLA4_positive represented antiCTLA4 therapy; PD1_positive represented antiPD-1/PD-L1 therapy. m6A = N6-methyladenosine.

To explore the role of the m^6^Ascore in clinical applications, Kaplan–Meier analysis was performed to investigate the prognostic value of the m^6^Ascore in patients at different stages. Patients with a low m^6^Ascore significantly associated with favorable prognosis in both stage I to II and stage III to IV (Fig. 7A and B). Patients with high tumor mutation burden (TMB) are reported more likely to benefit from immune checkpoint inhibitors (ICIs) therapy.^[27]^ The distribution of somatic mutations illustrated that TMB was more extensive in the low m^6^Ascore group (94.76% vs 88.11%, Fig. 7C–D). Furthermore, survival analysis confirmed that the prognosis of patients with higher TMB was favorable, and the high-TMB + low-m^6^Ascore group was superior to others for survival (Fig. 7E–F). The expression of PD-L1 in the low m^6^Ascore group was higher than that of high m^6^Ascore group (Fig. 7G). We then investigated the association between the IPS and the low/high m^6^Ascore group. Based on the IPS, we observed the difference of immunotherapy scores between high and low m^6^Ascore groups. We found that in the CTLA4_negative + PD-1_positive and CTLA4_positive + PD-1_positive types, the IPS of the low m^6^Ascore group was significantly higher than that of high m^6^Ascore group (Fig. 7I and K). In the CTLA4_negative + PD-1_negative and CTLA4_positive + PD-1_negative types, the median IPS for these 2 groups was nearly matching (Fig. 7H and J).

## 4. Discussion

The TME plays an important role in the occurrence and development of tumorigenesis, and epigenetic modification is a crucial mechanism in tumorigenesis.^[12]^ The m^6^A modification, a common epigenetic modification in RNA methylation, plays a pivotal role in TME and m^6^A regulators are key characters in the biological function and processing of m^6^A methylation modification.^[28–31]^

Here, we screened out 19 acknowledged m^6^A regulators and identified 14 regulators associated with prognosis of melanoma. We considered that the genetic variation of m^6^A regulators could affect their expression in melanoma, and suspected that disturbance in the expression of m^6^A regulators might function in the occurrence and progression of melanoma.

Based on 14 m^6^A regulators, 3 m^6^A methylation modification patterns were identified that correlated with different immune infiltration characteristics. The m^6^Acluster A was characterized by immune-related pathways, summarized as an immune-inflamed phenotype. We speculate that m^6^Acluster A is mainly related to immune activation. While m^6^Acluster B was not connected with immunity, the enriched pathways are often dysregulated in cancer. The m^6^Acluster C was characterized by noninflammatory pathways such as metabolism and neuroendocrine features, corresponding to an immune-desert phenotype. We further proved that the m^6^Acluster-A had more abundant immune cell infiltration than the other 2 clusters and correlated with a favorable prognosis. These 3 distinct m^6^A modification patterns, presenting significant differences in biological function, signaling pathways, and prognosis, also differed in immune cell infiltration and immunogenicity. In summary, we view the m^6^Acluster A as an inflamed tumor (hot tumor), while cluster B and C are noninflamed tumors (cold tumor). Generally, inflamed tumors are more sensitive to immune checkpoint inhibitors, and switching the tumor phenotype from cold to hot could enlarge the application of immunotherapies.^[32,33]^ Our results showed that these 3 m^6^A modification patterns were linked to distinct immune characteristics, and patients in m^6^Acluster-A with high immunogenicity might benefit more from immunotherapies.

Further, DEGs identified from the 3 patterns were enriched in m^6^A modification and immune-related pathways. Based on DEGs with prognostic value for melanoma, 3 genomic subtypes were identified, consistent with the results of m^6^A modification clustering and were termed as gene clusters A-C. These new genomic subtypes were likely connected to different immune characteristics in melanoma because the expression of the 14 m^6^A regulators in the 3 gene clusters were different, as well as the mRNA transcriptome in the 3 m^6^A modifications. We considered these DEGs as m^6^A signature genes. Furthermore, because of individual tumor heterogeneity and complexity, we constructed a score system to quantify different m^6^A modification patterns of individual tumor, named the m^6^Ascore. As a result, the m^6^Acluster A characterized as an immune-inflamed phenotype exhibited a lower m^6^Ascore, and the other clusters considered as a noninflamed phenotype showed a higher m^6^Ascore. This score system classified patients into 2 groups, and the prognosis of low m6Asocre group was favorable. Moreover, the same results presented in patients with different stage tumors, and patients with alive status showed a lower m^6^Ascore. These observations imply that the m^6^Ascore is a new marker that can predict the prognosis of melanoma.

Melanoma is associated with a tremendous number of somatic genetic alterations.^[34,35]^ Theoretically, the more mutations, the tumor is more likely to generate neoantigens and to have a better response to ICIs.^[27]^ The distribution of somatic mutations illustrated that TMB was more extensive in the low m^6^Ascore group. We deduced that the TMB of the low m^6^Ascore group was higher than that of the high m^6^Ascore group. Studies have shown that high TMB was more sensitive to immunotherapy, and the prognosis of patients with high TMB was favorable after receiving ICIs.^[36,37]^ The K-M curve in our study also showed that patients with high TMB had a better survival prognosis. Moreover, the combined m^6^Ascore and TMB survival analysis showed that the prognosis of high-TMB + low-m^6^Ascore group was more favorable than other groups. Based on these results, we conclude that patients with a low m^6^Ascore and high TMB would be better responders to immunotherapies; the survival prediction was more favorable as well.

Melanoma is an immunogenic tumor and is highly responsive to immunotherapy. However, the clinical application of PD-1/PD-L1 is limited because of individual heterogeneity and high cost of medical treatment. Thus, predictors of efficacy to PD-1/PD-L1 are vital for selection of subjects for immunotherapy, including PD-L1 expression, TMB, TILs, mismatch-repair (MMR) deficiency, and microsatellite instability (MSI).^[38]^ The expression of PD-L1 in the low m^6^Ascore group was higher than that of high score group. Further analyses revealed that the m^6^Ascore system showed a negative correlation with numerous immune cells, and the low m^6^Ascore group presented with an abundant enrichment in TILs, which implied that low m^6^Ascore group had a strong immune infiltration. Consideration of expression of PD-L1 and TILs in low-m^6^Ascore group, we speculated that m^6^Ascore could predict the efficacy of PD-1/PD-L1 inhibitors, and patients with melanoma in low m^6^Ascore group were probably the optimal candidates for immunotherapy.

The immunophenotypic score (IPS) is based on the expression of important components of tumor immunity, including MHC molecules, immunomodulatory molecules, effector cells and suppressor cells. We found that in the Cytotoxic T lymphocyte-associated protein-4 (CTLA4)_negative + PD-1_positive and CTLA4_positive + PD-1_positive types, the low m^6^Ascore group exhibited significant higher IPS. CTLA-4 and PD-1 are immune checkpoint inhibitors that have been identified as antibody immunotherapy targets for the treatment of melanoma, and the combination of CTLA4 and PD-1 was shown to significantly improve the survival of patients with metastatic melanoma.^[39–42]^ The analysis of combinations of antiPD-1 and antiCTLA-4 also revealed that the low m^6^Ascore group showed a higher positive response to antiPD-1 therapy and combination therapy of antiCTLA4 and antiPD-1. Our findings predicted that the m^6^Ascore correlates with the response to immunotherapy, and the low m^6^Ascore is more suitable for immunotherapy. Moreover, patients in the low m^6^Ascore group showed a higher positive response to antiPD-1 therapy and combination therapy of antiCTLA4 and antiPD-1.

Taken together, in this study, we determined 3 m^6^A modification patterns with different immune characteristics in melanoma patients. By considering the heterogeneity and complexity of individual tumor, we constructed a score system (m^6^Ascore) to quantify the m^6^A methylation modification, which could be an independent prognostic biomarker to predict patients’ survival. The m^6^Ascore can also be considered a novel biomarker that might be used an effective predictive strategy for immunotherapy.

This study also has some limitations. The m^6^A regulators involved in our study were the recognized from the previous literature, however, we might ignore some unknown m^6^A regulators. A set of recently discovered regulators must be integrated into the system to enhance the precision of the m^6^A patterns. Meanwhile, the interaction and cooperation of m^6^A regulators remain unknown, and the inside mechanism of m^6^A modification in regulating the immune infiltration of melanoma needs to be further explored. Additional research is needed to evaluate the effectiveness of m^6^Ascore in forecasting the reaction to ICIs in more extensive clinical trials, and to investigate the connections among these m^6^A regulators in immune response.

## 5. Conclusions

In conclusion, 3 m^6^A modification patterns with different immune characteristics were identified in melanoma and a score system (m^6^Ascore) was constructed to quantify the m^6^A methylation modification patterns, which correlate with clinical prognosis and individual treatment.

## Author contributions

**Data curation:** Si Ouyang.

**Formal analysis:** Feixiang Wang, Peijie Chen, Kaixin Xiong, Yao Wang.

**Funding acquisition:** Yao Wang.

**Investigation:** Peijie Chen.

**Methodology:** Feixiang Wang, Peijie Chen, Kaixin Xiong, Zichuan Liu.

**Writing – review & editing:** Yao Wang.

## Supplementary Material

**Figure:**